# Minimizing Bleed-Through Effect in Medieval Manuscripts with Machine Learning and Robust Statistics

**DOI:** 10.3390/jimaging11050136

**Published:** 2025-04-28

**Authors:** Adriano Ettari, Massimo Brescia, Stefania Conte, Yahya Momtaz, Guido Russo

**Affiliations:** Department of Physics E. Pancini, University of Naples Federico II, Via Vicinale Cupa Cinthia, 26, 80126 Napoli, Italy; stefania.conte@unina.it (S.C.); yahya.momtaz@unina.it (Y.M.)

**Keywords:** ancient documents restoration, bleed-through removing, document segmentation, blind method, neural networks, artificial intelligence

## Abstract

Over the last decades, plenty of ancient manuscripts have been digitized all over the world, and particularly in Europe. The fruition of these huge digital archives is often limited by the bleed-through effect due to the acid nature of the inks used, resulting in very noisy images. Several authors have recently worked on bleed-through removal, using different approaches. With the aim of developing a bleed-through removal tool, capable of batch application on a large number of images, of the order of hundred thousands, we used machine learning and robust statistical methods with four different methods, and applied them to two medieval manuscripts. The methods used are (i) non-local means (NLM); (ii) Gaussian mixture models (GMMs); (iii) biweight estimation; and (iv) Gaussian blur. The application of these methods to the two quoted manuscripts shows that these methods are, in general, quite effective in bleed-through removal, but the selection of the method has to be performed according to the characteristics of the manuscript, e.g., if there is no ink fading and the difference between bleed-through pixels and the foreground text is clear, we can use a stronger model without the risk of losing important information. Conversely, if the distinction between bleed-through and foreground pixels is less pronounced, it is better to use a weaker model to preserve useful details.

## 1. Introduction

**Figure 1:**
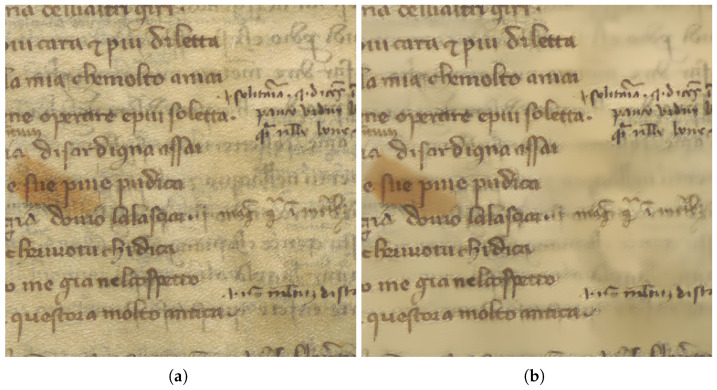
(**a**) The input image. (**b**) The cleaned image.

Ancient manuscripts suffer from aging problems, due to chemical agents, humidity, and other factors, all of which tend to diminish the document readability. The information contained in these manuscripts, however, is very important for the knowledge of the culture of those times, therefore it is important to try to increase the readability of manuscripts, and this can be achieved through digital technologies. The digitization process of ancient manuscripts being carried out at several cultural institutions has the result of stopping the aging problems at a certain time by acquiring a precise digital reproduction at a fixed date. Moreover, image processing on the digitized images can significantly improve the results, by transforming the acquired images into new images, more like the document as it was at the time of its creation. The result is a digitized manuscript in both the original scan and in the digitally improved scan, thus allowing different readers, from high school students to university scholars to researchers, to benefit from the information contained in the manuscripts. In this paper, we propose new methods for bleed-through removal (Figure 1), which is one of the most common and invalidating effects that involves documents that have been written or printed on both sides of the page. Its cause is the ink seeping from each side of the page onto the other. This type of degradation affects the appearance and the readability of the page. The code is available on Github (https://github.com/AdrianoEttari/bleed-through-cleaner, accessed on 17 April 2025).

This paper is organized as follows. In Section 2, we present the state-of-the-art methods addressing the bleed-through cleaning problem. Section 3 introduces the two codices we worked on. Section 4 gives a chemical explanation about bleed-through. Section 5 is devoted to the explanation of the methods we use to segment the images and to reduce the bleed-through. Section 6 gives some insight about the experiments we conducted on the two codices described in Section 3, as well as other types of data, using the methods outlined in Section 5.

## 2. State of the Art

The most effective methods for bleed-through reduction exploit information from both sides (recto and verso) of the page and they are called not-blind methods. They differ from the blind ones, which are less effective since they do not leverage this double information. However, these double pieces of information of the page are heavily dependent on the way the pages were scanned. One of the main requirements of the methods that exploit the information of the recto and verso sides of the page is that both sides of a scanned page are perfectly aligned, that is, each pair of coordinates *(i,j)* of a pixel on one side perfectly matches the same pair of indices *(i,j)* on the other. The alignment of the recto and verso sides of a page is called registration. An example of such a method is described by Hanif et al., 2018 [1], which exploits the registration of the pages to reduce the bleed-through.

Another interesting not-blind work was performed by Dubois et al., 2001 [2]. In this work, the authors not only showed how to identify and remove the bleed-through on a registered document by leveraging the recto and verso sides of the pages, but also presented a way to register the sides, which is the most complicated task.

A different approach based on a not-blind technique was described in Savino et al., 2019 [3] and 2024 [4]. Its peculiarity was the use of a local alignment of recto and verso pages, thus resulting in a third category, between the blind and not-blind ones, with advantages being applied to both of them. The main assumption was that the deformation between the recto and verso sides, although not identified by a unique geometrical transformation, is locally rigid. Therefore, by splitting the image into small patches, we can assume that their misalignment can be approximated by a rigid translation. The authors make use of the cross-correlation and the Fourier transform to calculate the local shift, and once the alignment is performed they apply a supervised model for classifying the pixels into foreground, background, and bleed-through. Their approach uses two clean (in terms of bleed-through) patches of the same image, combined to simulate the bleed-through effect. The model employs an ink penetration parameter to control the intensity of the ink from one patch over the other, effectively determining the strength of the bleed-through effect. Using this method, the authors were able to generate numerous bleed-through-affected patches with varying levels of intensity from just two original patches. This technique, combined with their innovative methods for locally registering images and inpainting the bleed-through areas, enables the creation of clean images using a simple and shallow neural network.

Hu et al. [5] presented an interesting workflow of a blind method for the bleed-through cleaning. They began by extracting global and local features using the Gaussian mixture model (GMM). Then, they employed the Extreme Learning Machine (ELM) [6], which is a neural network algorithm with a single hidden layer that computes output weights analytically, to classify pixels into foreground, background, and bleed-through. Finally, they applied image post-processing and performed simple inpainting of the bleed-through pixels using the mean value of the background Gaussian distribution in GMM, effectively removing the bleed-through while preserving the original image’s style.

A more probabilistic driven approach of removing the bleed-through effect on ancient documents is presented by Sun et al. [7]. In their work, they performed a coarse classification using component-wise Conditional Probability Distribution (CPD) modeling (i.e., Logistic regression and Gaussian mixture models), which is then refined by a Conditional Random Field (CRF) [8]. CRF is used as a probabilistic model that considers both the pixel’s individual features and its relationship with neighboring pixels. This helps in accurately segmenting text, background, and bleed-through areas. The bleed-through areas identified by CRF are then substituted using Random-Fill inpainting, which consists of replacing a bleed-through pixel with a background pixel randomly chosen within the local window.

Usually, the scanned documents are converted to png or jpeg images with three channels (red, green, and blue). However, it is also possible to use hyperspectral images as performed by da Costa Rocha et al., 2018 [9]. The contribution of a multi-band representation of an image may help identifying the bleed-through areas because under some specific wavelengths, the seeping ink may tend to fade. They fit a k-means algorithm on 160 bands of the hyperspectral images to segment the bleed-through and then they accurately classified the results using the Canny edge detection method on a band where the transparency is particularly different from other elements on the page.

Hanif et al., 2023 [10] explored alternative color spaces beyond the conventional RGB to enhance segmentation results. The motivation behind this approach is that combining different color spaces can improve segmentation performance, since object classes are better represented in specific color spaces. In their study, they integrated the RGB, HIS, and CIE color spaces from the same ancient document page and reduced the combined channels to three using Principal Component Analysis (PCA). Subsequently, they applied a Gaussian mixture model (GMM) to classify pixels into foreground, background, and degradation categories. Finally, they employed a texture inpainting method based on Gaussian conditional simulation to restore the degraded pixels.

Typically, the problem of bleed-through cleaning is addressed using unsupervised methods. However, Villegas et al., 2014 [11] successfully implemented a supervised approach. They labeled region samples as either clean text or bleed-through and identified a parametric function that maximized the variance of the clean text while minimizing the variance of the bleed-through. Stronger parameter settings produce cleaner images but may also smooth or remove the clean text and background texture.

## 3. The Codices Analyzed

We worked on two codices, the codex Filippino of the Girolamini Library in Naples, Italy, and the codex Pluteo of the Laurentian Medici Library in Florence, Italy. The Filippino Codex 2.16 is a parchment codex measuring 276 × 191 mm, with paper flyleaves, consisting of ff. II + 239 + II. It features a modern binding (1600–1700) in brown leather decorated with gold on cardboard plates. The codex is made up of binions, ternions, and quaternions, with a break in the quire between the canticles, but the predominant quire is the quaternion. Corresponding to the caesuras of the canticles, ff. 85v–86r and 165v–166r are blank. It is missing some leaves: ff. 153 and 154 (Purgatory, XXVIII 124–48; XXIX 1–105) and a leaf between ff. 197 and 198 (Paradise, XV 22–87) have been lost. In good condition, it has a triple cartulation: a modern numbering in Arabic numerals executed in pencil in the upper external margin, sometimes lower (1–239), a modern numbering in pencil in Arabic numerals in the center of the lower margin (1–239), and traces of an ancient numbering in Roman numerals executed in ink in the upper external margin (1–236). The writing is monocolumnar with protruding tercet initials and references in the center of the lower margin. The codex shows dry ruling and is enriched by historiated initials of the first two canticles, 146 framed miniatures, and two full-page diagrams of Purgatory and Paradise. From a paleographic point of view, it is a writing in chancery and notarial cursive written by various copyists; the manuscript was produced in Italy between the 1450s and 1460s. It consists of numerous interlinear and marginal glosses and annotations. As for the history of the manuscript, it was first part of the collection of the Neapolitan Poderico family, as demonstrated by the coat of arms present on c. 1r, then from Giuseppe Valletta’s collection, and was finally passed in 1726 to the Girolamini Library.

The Codex Pluteo 40.1 is a parchment codex measuring 250 × 345 mm, with parchment and paper flyleaves, consisting of cc. IV + 338 + III. It consists of a Medici binding dating back to 1571, characterized by wooden boards, a brown leather cover decorated with dry embossing, and metal elements, such as studs and cornerstones reproducing the Medici family coat of arms. At the caesurae of the canticles, cc. 111v–112r–112v and 210v are blank. In a sufficient state of conservation, it has a single page layout. A modern numbering in Arabic numerals is executed in pencil in the upper external margin (1–338); however, on f. 338 there are traces of a double ancient numbering in Arabic numerals executed in ink in the upper external margin. The writing is bicolumnar, sometimes with protruding tercet initials. The codex shows references in the center of the lower margin and a lead ruling. The codex is enriched by a full-page frontispiece, 102 drawings in the text columns, 201 decorated initials, and 18 diagrams. From a paleographic point of view, there is a bastard letter written by two copyists, one of whom is Gaspare Tommaso de Montone; the manuscript was produced in 1456. It is composed of glosses and marginal notes. Regarding the history of the manuscript, it comes from the private collection of the Medici family, whose original nucleus is identified in the 63 books owned by Cosimo the Elder; it found a place in the plutei (benches) at the opening of the Laurentian Medici Library, which took place in 1571 [12,13,14].

## 4. Factors of Parchment Deterioration: Iron Gall Inks

Parchment, as an organic compound, undergoes temporal deterioration. The amino acids that make up the basic unit of parchment, namely collagen, a fibrous protein present in animal tissues, are numerous and do not all have an identical structure; indeed, they are organized on different levels, each with its own specific bonds. Every single change at the molecular level that the parchment undergoes can lead to a modification of its mechanical properties, determined by internal factors, such as incorrect skinning or treatment during the manufacturing phase, a calcination phase that is too prolonged: the Philippine Code 2.16 of the Girolamini, for example, is characterized by a surface that is not perfectly straight, due to a too short application on the frame, which did not allow the collagen fibers to arrange themselves correctly. Instead, the Laurentian codex Pluteo 40.1, which has also been restored, finds not only brown stains on both sides of the sheet, but also gaps along the margins, caused by environmental factors, first and foremost humidity and temperature. In this case, this very hygroscopic organic material was subjected to a very high temperature, such as to modify the structure of the collagen protein, causing irreversible damage. Although parchment, unlike paper, does not incur acidity problems thanks to the treatments it is subjected to during manufacturing, it is always necessary to analyze the inks used and evaluate the effects linked to their composition. The ink used in the Middle Ages was based on vitriol, that is, ferrous sulfate, gall nuts (an excrescence caused by the action of insects on oaks and other types of plants) containing tannin, and gum arabic as a stabilizer–viscosifier. Ferrous sulfate penetrates the fibers of the support quite easily due to its solubility and over time undergoes transformations that lead it to the state of iron oxide. Given the positive results offered by ferrous sulfate with regard to erasure, it was thought to add increasingly greater quantities. Excess ferrous sulfate leads to an excess of Fe (II) ions, which are dangerous as they act as catalysts for oxidative degradation reactions. The interaction between the iron salt and the tannic and gallic acids produces hydrogen ions H^+^, which combine with the excess sulfate ions to form sulfury acid. Acid can migrate to the back of the overlying sheet, producing brown writing in the opposite direction as in front of a mirror: this phenomenon is known as bleed-through. The addition of pieces of iron to the ink was recommended to reduce acidity. Fortunately, parchment, unlike paper, does not suffer the destructive effects of acidity, since it has a sufficient alkaline reserve inside, deriving from the leather processing. It is treated in lime baths, and in the subsequent washing with water, not all the calcium hydroxide is eliminated but a part is transformed into calcium carbonate, which allows the parchment to neutralize any onset of acidity from whatever source it comes from. Furthermore, in the MAGIC project, realized in collaboration between the Department of humanistic studies and the Department of physics “Ettore Pancini” of the University of Naples “Federico II”, the Materials Optics Group of the Department of physics is contributing to the analysis of the state of conservation of inks, using Fourier transform infrared spectroscopy (FT-IR) techniques. Portable versions of spectroscopic characterization instruments have been purchased, to allow in situ analysis of the works, where the size or condition of the ancient volumes prevent their transportation or observation with traditional instruments. Considering that parchment materials, of organic origin, are affected by time by aging and degrading naturally and that it is not possible to erase stains caused by pathogenic agents without irreparably damaging the support, it is necessary to keep the conservation environment under constant control: among the objectives of the MAGIC project, there is the identification of the causes and procedures for monitoring the progress of degradation. However, as part of the project’s research activity, various artificial intelligence systems are being explored, to identify the most effective techniques for attenuating the effects of degradation, first of all, the bleed-through effect [15,16,17].

## 5. Methods

**Figure 2:**
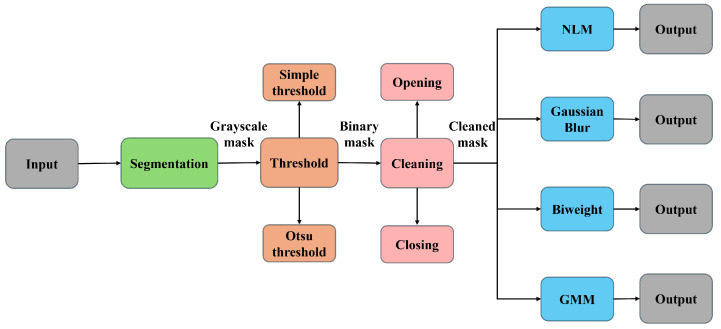
Diagram of the applied methods.

Models that use registered input data generally achieve better results. However, accurately registering the pages is challenging and highly dependent on the scanning process. Our bleed-through minimization approach works without the alignment technique because the scanning process did not take it into account. It is based on the segmentation of different components within a page, with a further denoising algorithm to detect and reject the bleed-through presence, preserving all the rest. Our workflow involves extracting the page from the image by removing all irrelevant elements (e.g., the desk on which the book rests). This is achieved using a page segmentation mask generated by the corresponding algorithm, along with thresholding, morphological cleaning, and largest-component extraction techniques. Next, we divide the filtered image into patches. For each patch, we generate a text segmentation mask, which is binarized using a thresholding technique, and an ornament segmentation mask, which undergoes further refinement through thresholding and morphological cleaning. We then combine these two masks, and inpaint the non-segmented areas (i.e., white regions) using non-local means (NLM) denoising [18]. We also experimented other inpainting methods, some of which outperformed NLM in specific cases. Figure 2 shows the main steps of our approach.

Before describing the application of this pipeline, in the following we introduce all methods and models shown in the pipeline block diagram.

### 5.1. Segmentation

The segmentation, the first step of our pipeline, consists of three neural network models, trained separately for each segmentation task. We segmented each image into three areas: the page (in the images there are elements not belonging to the document, like the table where the book stands on), the text, and the ornaments (i.e., all types of drawings within an image).

**Figure 3:**
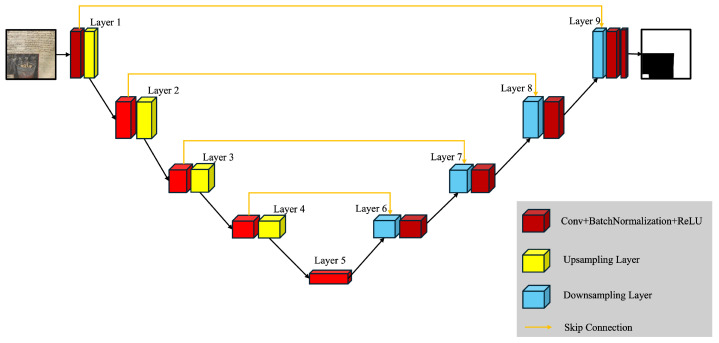
Example architecture of the UNet model.

The architecture of the neural network models is a simple UNet with 4 million parameters. The UNet [19] is a machine learning model mostly suitable for image segmentation. It has the structure of an autoencoder [20], with the addition of the skip connections that allow it to preserve fine-grained details throughout the model. They address the challenges inherent in deep learning for image segmentation, where both the global structure and fine details are crucial. Figure 3 shows an example of UNet architecture. The output of each segmentation model is a gray-scale image.

### 5.2. Thresholding

In image processing, thresholding is used to convert gray-scale images into binary images (i.e., all pixels above a threshold are assigned to a value of 1 and all pixels below this limit are assigned to a value of 0). Moreover, we can identify two types of threshold methods: global, which assigns a threshold value for the whole image, and local, which assigns different threshold values across different regions of the image. In this work, we apply two types of threshold techniques: (i) a simple global thresholding, where a fixed threshold determines if a pixel is 0 or 1, on the text and the ornament segmentation; and (ii) an Otsu’s thresholding [21], which is a global method that minimizes intra-class variance and maximizes inter-class variance on the page segmentation.

### 5.3. Morphological Cleaning

**Figure 4:**
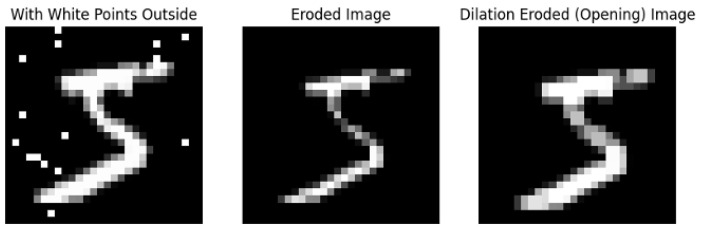
Example of opening operation (first erosion, then dilation).

Morphological operations are simple image processing techniques based on the shapes and structures within the image. They are performed on binary images and require two elements: the original image and the kernel that decides the nature of the operation. The morphological operations used in this work are opening and closing. The first is the application of erosion and dilation, in this order, and the latter is the opposite (first dilation and then erosion). The erosion considers a kernel full of “1” values and performs a sliding of this kernel on the image. It assigns a value 1 to a pixel *(i,j)* only if all the pixels in the window, centered at *(i,j)*, have value 1 (white pixels). This allows it to remove the isolated noisy pixels, but also it decreases the thickness of the foreground object as it is shown in the central image of Figure 4.

**Figure 5:**
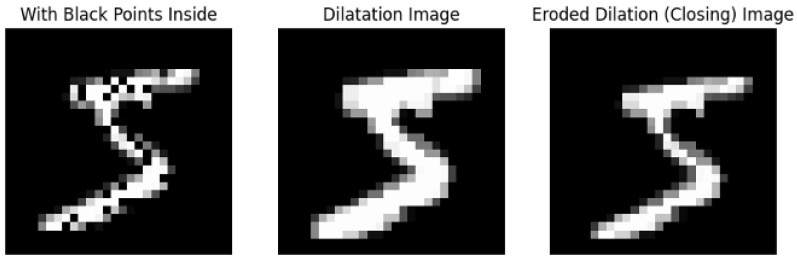
Example of closing operation (first dilation, then erosion).

The dilation does almost the same thing, but it assigns value 1 to a pixel *(i,j)* only if at least one pixel in the window, centered at *(i,j)*, has value 1 (white pixels). It “fills the gaps” in the foreground object and increases its thickness, as it is shown in the central image of Figure 5.

**Figure 6:**
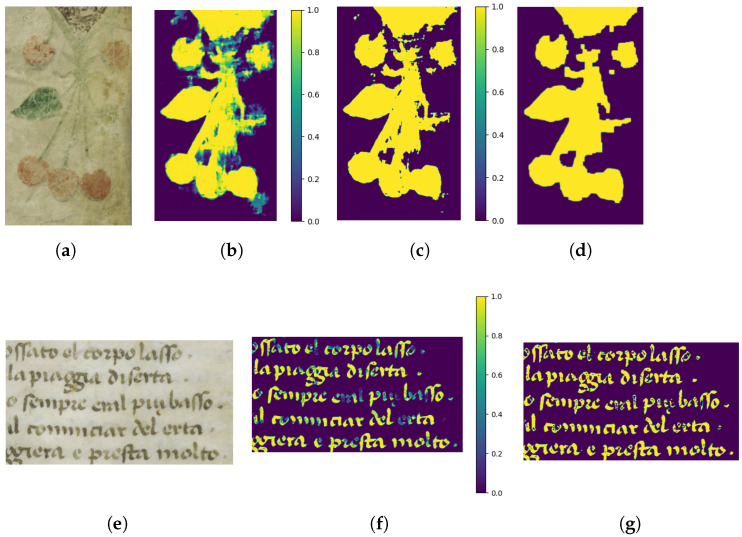
(**a**) Input image. (**b**) Gray-scale mask obtained through segmentation. (**c**) Binary mask obtained by applying a simple threshold on the gray-scale image. (**d**) Binary mask, cleaned through opening and closing morphological operations. (**e**) Input image. (**f**) Gray-scale mask obtained through segmentation. (**g**) Binary mask obtained by applying a simple threshold on the gray-scale image.

Figure 6 shows the steps to get a cleaned binary mask in the case of an ornament (first row) and in the case of a text (second row). In the case of text we don’t use any morphological operation.

### 5.4. Non-Local Means Denoising

The non-local means (NLM) denoising [18] technique is particularly effective in denoising while preserving fine details and textures in an image. The core idea of this method starts from the classical denoising mechanism, consisting of replacing the intensity value of a pixel with an average of the intensities of nearby pixels, and by applying the well-known variance law in probability theory, for which if *n* pixels are averaged, the standard deviation of the average pixel is divided by $\sqrt{n}$. Thus, if we can find *n* pixels with almost the same true underlying intensity (i.e., except for small disturbances due to the presence of noise), by averaging them we can decrease the overall noise. The problem is where and how to find those pixels within the image. The NLM is able to solve this problem. In fact, the authors of the proposed method [18] state that “similar pixels have no reason to be close at all. It is therefore licit to scan a vast portion of the image in search of all the pixels that really resemble the pixel that one wants to denoise”. For each pixel *i*, the algorithm compares its surrounding patch $P_{i}$ with all the other patches $P_{j}$ (having the same size) centered on pixels *j* within a search window, whose size is significantly bigger than the size of the patches *P*:

(1) $$\hat{I}\left(i\right)=\frac{\sum_{j}I\left(j\right)\cdot w\left(i,j\right)}{\sum_{j}w\left(i,j\right)}$$

(2) $$w\left(i,j\right)=e^{-\frac{\left|\right|P_{i}-P_{j}{\left|\right|}^{2}}{h^{2}}}$$

The following definitions are used above:

- I(j) is the intensity of the pixel *j*;
- $\hat{I}\left(i\right)$ is the weighted average intensity within the patch window of pixels centered on pixel *i*;
- $P_{i}$ is the image patch, i.e., a user-defined window of pixels centered on the pixel *i*;
- w(i,j) is the weight representing the similarity between the patches around pixels *i* and *j*;
- *h* is a smoothing parameter that controls how much the similarity between patches influences the averaging. A smaller *h* means that only very similar patches are averaged because the dissimilar ones will obtain small weights, while a larger *h* allows more distant patches to contribute to the denoised value.

The patch size around each pixel (e.g., 3×3 or 5×5 pixels, but always odd numbers because we need a central pixel) is important for the weight computation. Larger patches can capture more structural information, but increase the computational cost.

**Figure 7:**
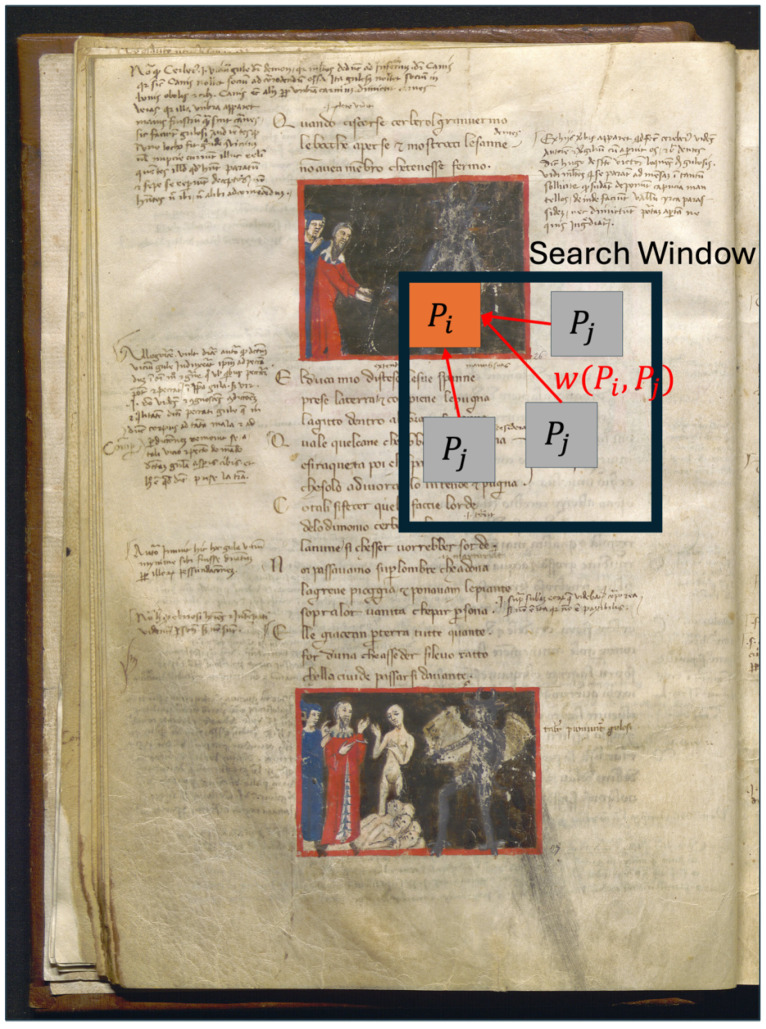
Weighted average of the pixels within a search window. The original image is from manuscript coded CF 2.16 (Filippino), page 14v, owned by Girolamini Library, Napoli, Italy. Image acquired by Dept. of Humanistic Studies, image processing by Dept. of Physics, University of Napoli Federico II, Italy.

As shown in Figure 7, the algorithm works by performing a weighted average of the pixels within a search window, with weights based on the similarity of the patches on which they are centered and where several search windows are used to cover the entire image. The size of the windows affects the performance and speed of the algorithm. This entire procedure is applied for each channel in the case of color images.

### 5.5. Biweight Estimation

In most cases, NLM represents a good choice in removing bleed-through and in returning more clean and good-looking images without losing the original appearance of the pages. However, some pages have some notes at the borders that are ruined and barely readable. In these cases, NLM performs a too-aggressive smoothing of the text, causing some notes to disappear. Although it would be possible to adjust the parameters of NLM in order to decrease the smoothing strength, we verified that the biweight robust estimation [22] works better in these situations.

Biweight is a robust statistical estimator used to measure the central tendency (such as mean or median) and the scale (such as variance or standard deviation) of a distribution. It is robust because it down-weights the contribution of extreme values, providing a more representative measure of the central tendency. The workflow of the biweight estimation is described in what follows:

1. Compute μ, which is the mean or an initial estimation of the central tendency of the image;
2. Compute $u_{i}$, which is a weighting factor, based on the scaled deviation of each data point from μ:
  (3) $$u_{i}=\frac{I(i)-\mu }{c\cdot MAD}$$
  The following definitions are used above:
  - I(i) is the intensity of the pixel *i*;
  - *MAD* is the Median Absolute Deviation of the pixels;
  - *c* is an empirical tuning constant, also known as cut-off point, which modulates the way to down-weight the image values that deviate from the central location. Smaller values of this constant make the biweight estimation more sensitive to outliers (it removes more outliers, becoming more robust), while larger values make the biweight more tolerant to outliers. As discussed in [23], the best balance of estimation efficiency was found for c=6.0, which makes the method able to include data up to 4σ (σ is the standard deviation of the image pixel distribution) from the central position.
3. If the weighting factor $u_{i}$ is greater than 1, then the weight $w_{i}$ of the pixel *i* will not be updated, otherwise it obtains a value depending on $u_{i}$:
  (4) $$\left\{\begin{array}{c} if|u_{i}|>1\to w_{i}=0\\ else\to w_{i}={\left(1-u_{i}^{2}\right)}^{2} \end{array}\right.\;$$
4. Finally, compute the biweight location:
  (5) $$T=\mu +\frac{\sum_{i=1}^{n}w_{i}\left(I\left(i\right)-\mu \right)}{\sum_{i=1}^{n}w_{i}}$$

This new value is close to the original mean but has reduced sensitivity to outliers. The biweight location value replaces the pixel values identified as outliers by a technique known as sigma clipping [24], which is a statistical method mostly used in astrophysical images characterized by a high resolution and a low signal-to-noise ratio, able to iteratively exclude pixels that significantly deviate from the median in an image. This method involves setting an arbitrary number of iterations and a threshold value, *k*, then applying the following iterative procedure:

1. Calculate the standard deviation (σ) and median (*m*) of the pixel distribution;
2. Remove all pixels that are smaller or larger than m±kσ;
3. Go back to step 1, unless the selected exit criteria is reached, where the exit criteria are based on two possible conditions:
  - After reaching a user-defined amount of iterations;
  - When the new measured standard deviation σ is within a certain tolerance level of the old one (i.e., the standard deviation measured at previous iteration). Here, the tolerance level is defined by: $\frac{\sigma_{old}-\sigma_{new}}{\sigma_{new}}<1$, where at each clipping iteration the new value of standard deviation of pixels is always equal or less than the previous one, (i.e., $\sigma_{new}\leq \sigma_{old}$).

The sigma clipping in our case was applied by flagging at each iteration the pixels excluded by the method (i.e., those considered as outliers) and by replacing them with the calculated biweight location value. All the other pixels passing the sigma clipping iteration were left unchanged.

### 5.6. Gaussian Mixture Models

A GMM [25] is an unsupervised machine learning method based on the assumption that all pixels come from a mixture of Gaussian distributions with unknown parameters. They are parametric generative models that attempt to learn the true pixel distribution. GMM demands the choice of a number of clusters *k* as an input to the learning algorithm and can learn clusters with arbitrary shape, by providing probabilities that relate each pixel with a given cluster. In other words, GMM allows for any pixel to belong to more than one cluster, with a level of uncertainty and it learns the probabilities of that pixel to belong to each cluster *k*.

In general, GMM tries to learn each cluster as a different Gaussian distribution. It assumes the data are generated from a limited mixture of Gaussians. Given an arbitrary number of clusters *k*, GMM attempts to learn three parameters for each cluster *k*, mean, variance, and a scaling term, assuming that each cluster is represented by an individual Gaussian distribution. To learn such parameters, GMMs use the Expectation Maximization (EM) algorithm to optimize the maximum likelihood and the Bayes Theorem to calculate the probability of a given pixel to belong to each cluster. In practice, in the univariate case, we try to model a set of data patterns (for instance groups of image pixels) using a mixture of Gaussian prior distributions on the estimates, resulting as follows:

(6) $$P\left(x\right)=\sum_{i=1}^{k}\phi_{i}N\left(\mu_{i},\sigma_{i}^{2}\right)$$

where $\phi_{i}$ are weights of a single normal distribution $N(\mu_{i},\sigma_{i}^{2})$ defined by its mean $\mu_{i}$ and variance $\sigma_{i}^{2}$ for each of the *k* clusters.

Thanks to the Bayesian estimation rule, the weights $\phi_{i}$ are multiplied to the known distribution $P(x|\mu_{i},\sigma_{i}^{2})$ of pixels conditioned by the mean and variance parameters to estimate. In this case, the posterior distribution $P(\mu_{i},\sigma_{i}^{2}|x)$ becomes a Gaussian mixture model defined by

(7) $$P\left(\mu_{i},\sigma_{i}^{2}|x\right)=\sum_{i=1}^{k}\phi_{i}^{*}N\left(\mu_{i}^{*},\sigma_{i}^{2*}\right)$$

where the symbol * indicates the parameters and weights to be estimated by the EM algorithm. In a multivariate case, the same procedure is followed, by just replacing variances with the covariance matrices. As the initial condition of the EM algorithm, random values are assigned to the parameters of Equation (7). Then, the maximum likelihood optimization loop starts by performing the expectation step followed by its maximization. In the expectation step, we calculate the likelihood of each pixel using the actual parameters. This corresponds to calculate for each cluster the Probability Density Function (PDF) of the pixel distribution using the current estimated mean and variance, which at the beginning of the optimization loop are mere random guesses, using the known Gaussian PDF function:

(8) $$f\left(x|\mu ,\sigma^{2}\right)=\frac{1}{\sqrt{2\pi \sigma^{2}}}e^{-\frac{{\left(x-\mu \right)}^{2}}{2\sigma^{2}}}$$

Equation (8) is hence used in the expectation step to calculate the likelihood of each pixel $x_{i}$ belonging to the *j*-th cluster, given *n* total pixels and *k* clusters:

(9) $$L_{ij}=\frac{f\left(x_{i}|\mu_{j},\sigma_{j}^{2}\right)\phi_{j}}{\sum_{c=1}^{k}f\left(x_{i}|\mu_{c},\sigma_{c}^{2}\right)\phi_{c}}\;i=1,\ldots ,n\;j=1,\ldots ,k$$

It is worth noting that the parameters $\phi_{j}$ act as our prior beliefs that a pixel was drawn from one of the Gaussians we are optimizing (i.e., the weights of the GMM). Since we do not have any additional information to prefer a Gaussian over the others, we simply start by guessing an equal probability that a pixel would come from each Gaussian. During the optimization process however, the three parameters are iteratively refined until convergence. This refinement is performed during the maximization step, where the parameters are re-estimated using the following expressions to update the expectation components, using the likelihood obtained by Equation (9):

(10) $$\mu_{j}=\frac{\sum_{i=1}^{n}\left(L_{ij}x_{i}\right)}{\sum_{i=1}^{n}L_{ij}}\;i=1,\ldots ,n$$

(11) $$\phi_{j}=\frac{1}{n}\sum_{i=1}^{n}L_{ij}\;i=1,\ldots ,n$$

(12) $$\sigma_{j}^{2}=\frac{\sum_{i=1}^{n}L_{ij}{\left(x_{i}-\mu_{j}\right)}^{2}}{\sum_{i=1}^{n}L_{ij}}\;i=1,\ldots ,n$$

The two steps of expectation and maximization are iterated until convergence. The GMM returns soft clustering (i.e., each pixel obtains the probability of belonging to each cluster). Moreover, it can learn clusters of any ellipsoidal shape, size, density, and orientation. One of its main drawbacks is the need of choosing a specific number of components, but we can leverage the Akaike Information Criterion [26] and the Bayesian Information Criterion [27] to choose the best number of clusters for a specific use case (notice that other clustering techniques like K-Nearest Neighbor [28] use the Silhouette coefficient [29] and the Elbow method [30] to choose the best number of clusters, but these methods work better if the clusters are spherical). The choice of the EM algorithm was motivated by its frequent use in the literature. Therefore, an attempt with this method was useful to compare its performance with respect to our proposed alternative using NLM, which obtains better results. The internal setup of the EM was based on default settings of GaussianMixture function present in the sklearn library. For instance, the EM stopping criterion is based on the threshold of 0.001; the number of iterations is set to 100; and the weights are initialized using K-means.

### 5.7. Gaussian Blur

**Figure 8:**
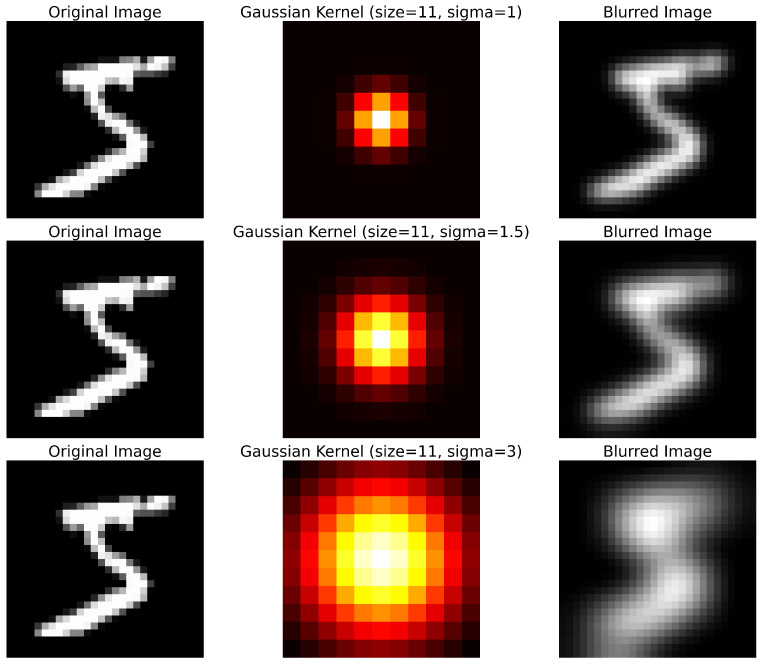
Application of three Gaussian kernels on the same image.

Like NLM, Gaussian blur performs pixel smoothing. The difference is that NLM averages pixels that have similar values, while Gaussian blur averages pixels that are in the same neighborhood, for which it is reasonable to consider a kernel with normally distributed values. Its shape and standard deviation are the only parameters that the user must specify. As the kernel and standard deviation increase, the image will appear smoother, but with an increase in computational cost. We perform a sliding window of this kernel on the image and the result is the smoothed image shown in Figure 8.

## 6. Experiments and Results

In this section, the results will be presented both qualitatively and quantitatively. The latter can be challenging to evaluate, as good statistics do not always guarantee good results. Therefore, we evaluate the foreground and the background errors in the predicted segmentation masks, as performed in [3,4] and the metrics used in the DIBCO competitions like in [31,32]. Therefore, we are not going to validate the inpainting part (NLM) quantitatively.

### 6.1. Workflow

**Figure 9:**
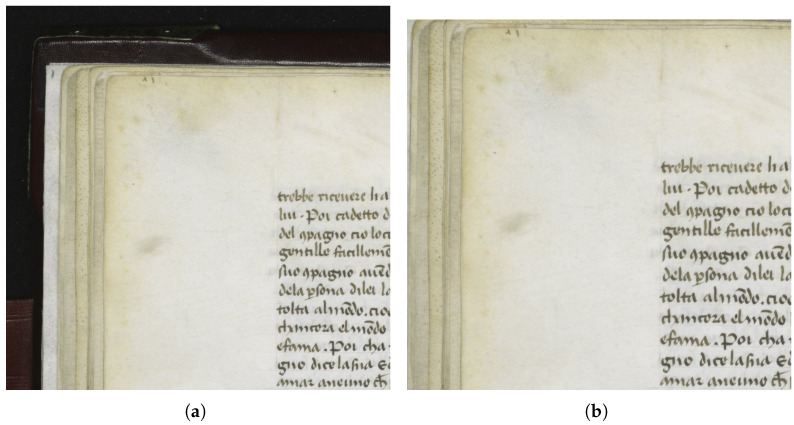
(**a**) A crop of the input image. (**b**) A crop of the page filtered image. In this example the shape of the page, where the crop comes from, moves from (2415, 3374) to (2270, 3265), since the page filtering model discards 736,660 pixels belonging to useless parts of the image.

The workflow of the whole bleed-through removal process is described in the following. First, a supervised model for page filtering is trained with the aim of cutting out all the border regions in the images that are not part of the document and that contribute to making the other segmentation models slower and with poorer performance. The page segmentation model returns a gray-scale mask that is then binarized via Otsu thresholding and cleaned via morphological transformations, specifically aperture filtering and maximum white area selection. We then use this cleaned binary mask to make the filtering of the page whose result will be called hereafter “page filtered image”. To train the segmentation model we use the dataset proposed in the paper [33]. The pages are resized to $6\times 10^{5}$ pixels and their aspect ratio is kept as suggested in the paper [34]. The page segmentation algorithm was trained with a batch size of 1 (because the pages are not split into patches and so the algorithm has input images of different shapes), a learning rate of 0.0002, Binary cross-entropy loss, Adam optimizer, and 20 epochs, completing in approximately 20 h on three NVIDIA V100 GPUs. Figure 9 shows an image before and after the application of this model.

**Figure 10:**
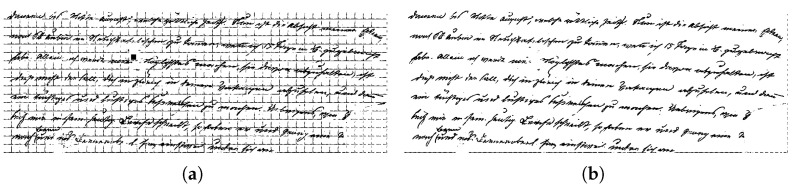
(**a**) The binary mask built without aggregation sampling. It has some square shapes inside, which is why it is called the chessboard effect. (**b**) The binary mask built with aggregation sampling.

Second, we split the page filtered image into overlapping patches with the aggregation sampling technique proposed in the paper [35], which avoids the chessboard effect in the reconstructed image. Figure 10 shows how aggregation sampling improves the building of the binary mask.

These patches, together with the corresponding ornament masks, will be used to train the ornament segmentation algorithm. We split the images into patches because neural networks train more efficiently in parallel with smaller images and because the pages vary in shape whereas the patches are built with the same shape. The shape variation poses a coding challenge, as the data fed into the neural network must be structured in a data loader. The data loader requires a batch size parameter, which determines the number of images concatenated in the same batch. For this concatenation, all images in the batch need to have the same shape. These patches are input to the supervised ornament segmentation algorithm that is trained to return a gray-scale mask for each patch. We then combine the mask patches by averaging the overlapping parts using Gaussian filtering (a technique used in the aggregation sampling method). The reconstructed gray-scale mask is then binarized with a simple global thresholding technique and cleaned using the opening and closing morphological techniques. To train this segmentation algorithm we built the dataset by exploiting the graphical image annotation tool, Labelme (https://github.com/wkentaro/labelme, accessed on 17 April 2025). The dataset used to train the ornament segmentation model has 12,705 non-overlapping patches (the overlapping strategy is used just at inference time because during training we do not need to reconstruct the images), shaped 400 × 400 and extracted from 150 pages of the two ancient documents. The ornament segmentation algorithm was trained with a batch size of 16, a learning rate of 0.0002, Binary cross-entropy loss, Adam optimizer, and 500 epochs, completing in approximately twenty hours on three NVIDIA V100 GPUs.

**Figure 11:**
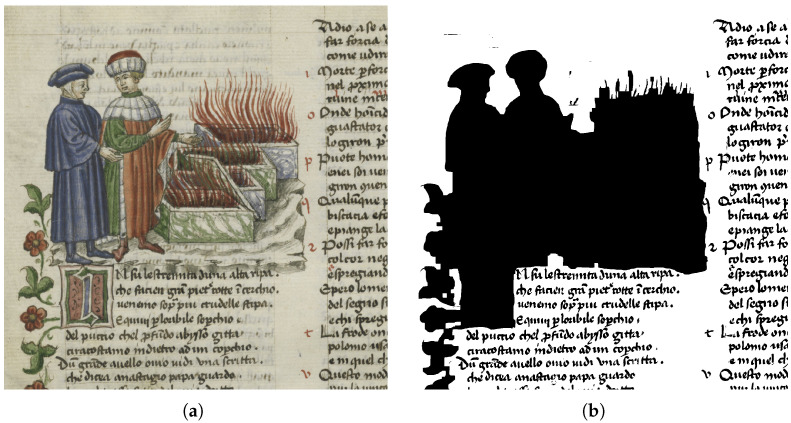
(**a**) Page filtered image. (**b**) Final binary mask.

Third, the page filtered image is split into patches like in the second step, to perform text segmentation with another supervised model, trained to perform such task. We then binarize the produced gray-scale mask with a simple thresholding mask. The dataset used to train this model has 17,200 non-overlapping patches shaped 400 × 400 that were extracted from the READ-BAD dataset proposed by Grüning et al., 2018 [36]. The text segmentation algorithm was trained with a batch size of 16, a learning rate of 0.0002, Binary cross-entropy loss, Adam optimizer, and 500 epochs, completing in approximately one day on three NVIDIA V100 GPUs. The text binary mask and the ornament binary mask are then summed up to obtain a final binary mask with pixels equal to 0 (black) segmented as either a text or an ornament and pixels equal to 1 (white) segmented as either background or bleed-through. In Figure 11, there is a page filtered image (left) and its final binary mask (right).

Finally, we perform inpainting using the non-local means denoising method on the entire filtered page image, using the OpenCV library function, fastNlMeansDenoisingColored (https://docs.opencv.org/4.x/d5/d69/tutorial_py_non_local_means.html, accessed on 17 April 2025). This function converts the image from RGB to CIELAB, which is a color space where each RGB image is converted to a LAB image where L is the luminance and A and B control the chrominance. The reason for this conversion is that this function obtains two *h* parameters, one for L and one for A, B. The *h* parameter of L controls the denoising strength for the gray-scale information and the *h* parameter of A and B (also called hColor) controls the denoising strength for the color channels. NLM is a versatile model. Instead of using two different models to minimize weak and strong bleed-through effects, we can simply adjust the denoising strength parameters (*h* and hColor) of NLM. If we aim to remove as much bleed-through as possible, we can increase the *h* and hColor values. However, this may result in a more artificial appearance and the potential loss of useful information, depending on the segmentation results. Conversely, keeping these parameters low still improves the image quality, but some bleed-through will remain visible. NLM has two additional parameters, patch size and window size, which are explained in Section 5.4 and illustrated in Figure 7. In the case of bleed-through reduction for manuscripts, the patch size should be chosen based on the font size of the text and, more generally, on the size of the features of the page, as NLM calculates weights based on patch similarity. Basically, each patch should comprehend at least a decent portion of a letter to correctly perform patch similarity. Performing similarity calculations across the entire image would be computationally expensive. Instead, we define a window size, restricting comparisons to patches within that window to efficiently compute patch similarities. We built two possible settings, where both filter strength and color filter strength refer to the h in the Equation (2) but relative to different channels: filter strength = 6, color filter strength = 20, patch size = 15, and window size = 35; and filter strength = 8, color filter strength = 20, patch size = 25, and window size = 55. The first setting is the “weak” one and the second setting is the “strong” one. The manuscripts we analyzed exhibit both ink fading and bleed-through issues, so we selected the “weak” NLM setting, ensuring that the image is not excessively smoothed. This prevents the loss of valuable information in cases where some pixels are mis-segmented. Instead, it enhances readability by softening the background and reducing bleed-through. Slight adjustments may be needed to achieve optimal results for different datasets.

The resulting image is free from bleed-through; however, the ornaments and the text have also been smoothed, which is undesirable as the goal is to smooth only the background and the bleed-through. To solve this issue, we replace the resulting page with the pixels of the page filtered image where the final mask is equal to 0 (black pixels). The result is a smooth page with the following effects:

- The text and the ornaments of the original page, as detected by the segmentation models, are represented exactly as they appear in the original image;
- The text and the ornaments of the original page that are not detected by the segmentation models are represented in a smoothed way relative to the original image;
- The bleed-through is smoothed and barely visible.

**Figure 12:**
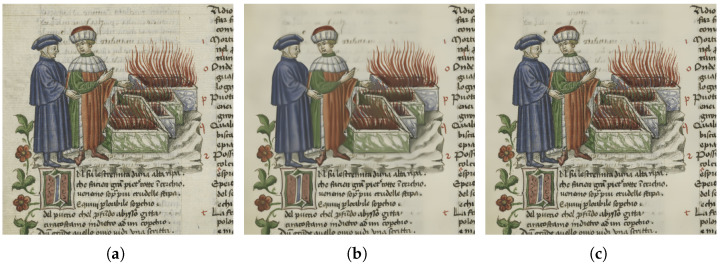
(**a**) The input image. (**b**) NLM applied on the input image. (**c**) NLM “modified” applied on the input image where “modified” means that the NLM is applied just where the segmentation mask is different to 0 (pixels classified as either background or bleed-through).

Figure 12 shows how NLM succeeds in minimizing bleed-through while preserving important information.

**Figure 13:**
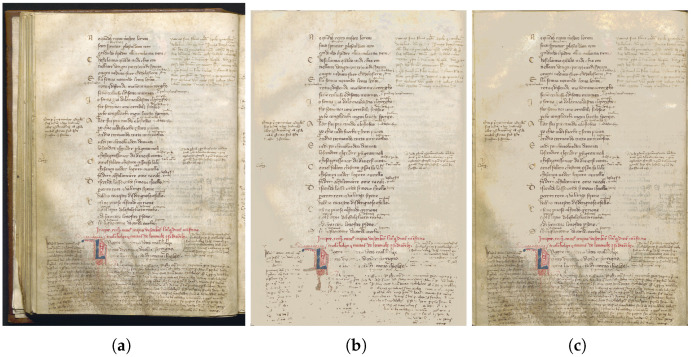
(**a**) Page filtered image. Original image is from manuscript coded CF 2.16 (Filippino), page 42v, owned by Girolamini Library, Napoli, Italy. Image acquired by Dept. of Humanistic Studies, image processing by Dep.t of Physics, University of Napoli Federico II, Italy. (**b**) The inpainting using biweight estimation with the initial mask. (**c**) The inpainting using biweight estimation with the inverted mask.

We noticed that the biweight robust estimation outperforms NLM when the border notes are barely visible, which is where the NLM may partially fail. In our application, sigma clipping discards parts of the image that do not belong to the page (e.g., the table where the document stands on), it removes the areas of the page that show significant signs of aging, and it also excludes the pixels where the ink from the front page faded. As a result, sigma clipping preserves only the portions of the pages in good condition, along with the bleed-through. This approach is problematic for our purposes, as we want the bleed-through to be treated as an outlier. However, we can work around this problem by inverting the output mask from sigma clipping, allowing us to replace the biweight location value in areas not initially flagged as outliers. Figure 13 demonstrates how the biweight location function operates and how we adapted it for our specific purposes.

**Figure 14:**
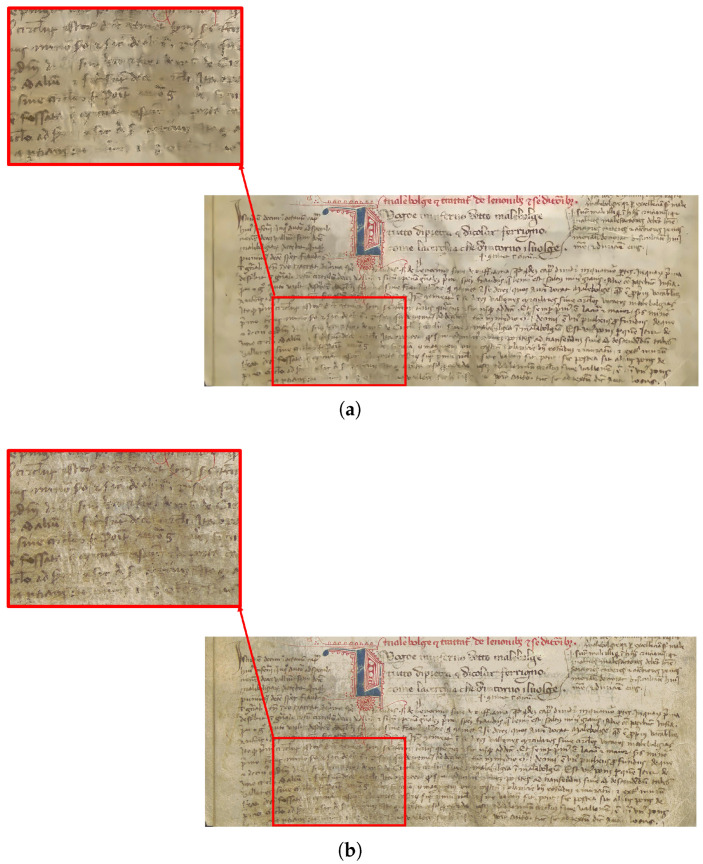
(**a**) The result of the NLM inpainting. (**b**) The result of the biweight inpainting. Even though, to someone not experienced in reading ancient documents, the two pages may appear identical, an expert in the field might, with time and effort, be able to read the page (**b**), while the page (**a**) remains unreadable.

As Figure 14 shows, in cases with important degradations different from bleed-through, NLM performs worse than biweight estimation.

Biweight cannot be the final choice because the resulting images are more “artificial” compared to the NLM ones and because it does not work well when there are variations in the light in the images.

To build the biweight estimation model, we used the default settings of the biweight_location and sigma_clip functions from the astropy.stats library in Python 3.10. However, we applied the inverted mask produced by the sigma_clip function, for the reasons outlined above.

NLM and biweight methods were not the only approaches we attempted. Prior to achieving success with these methods, we experimented with several others to address our objectives. For example, we tried the Gaussian mixture models (GMMs) in two ways:

- We fit a GMM on the original images. We set the number of Gaussian components to four (front text, background, borders of the page, and other different degradation patterns). Unfortunately, the model was not good enough to capture the differences between the bleed-through and the front text. The reason is probably that the distributions of the four classes, and especially the one of bleed-through, are very different among the pages and sometimes the thickness and the saturation of the bleed-through pixels are close to the one of the front text. Moreover, it takes really a long time to train a GMM on a considerable number of pages;
- We fit a GMM with three components (background, borders of the page, and other different degradation patterns), just on the pixels of the pages segmented as background/bleed-through by the segmentation models. The idea was to distinguish the clean background from the one affected by bleed-through. It works well on simple cases, but it still needs some inpainting techniques to replace the bleed-through pixels. This means that it needs significant computation time, the results are not optimal, and there is still the need for an inpainting technique to complete the task.

**Figure 15:**
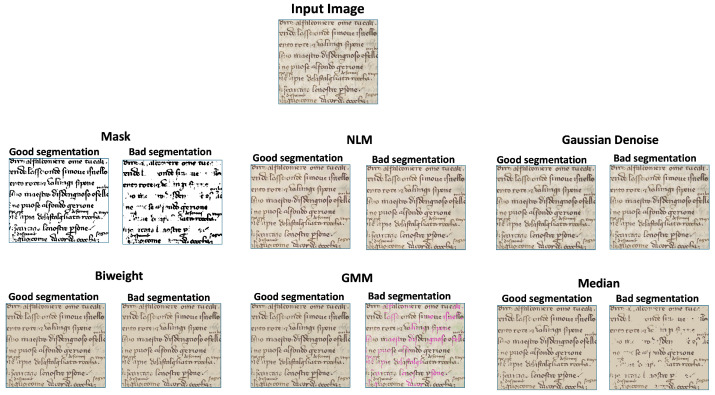
Example of how our methods work on a weak segmentation (**left column**) and on a good segmentation (**right column**). NLM, biweight, and GMM return nice results in both cases, whereas Gaussian denoising and median erase the good text that is not segmented. This approach does not diminish the value of a well-designed segmentation algorithm. Instead, it highlights that even on other ancient documents, where the text differs from that used to train the segmentation algorithm, the important pieces of information will not be deleted by these methods.

Before experimenting with NLM filtering, we tried to denoise the background with the application of a simple Gaussian filter. It averages pixels like NLM but focuses on those that are spatially close rather than focusing on those that have similar values. Achieving significant bleed-through reduction with Gaussian filtering requires a strong filter, which unfortunately results in an overly blurred and visually unappealing page. A key advantage of NLM (and sometimes also biweight) over the other methods is its effectiveness even when segmentation algorithms miss valuable text. Other methods would simply replace the missed text, potentially compromising the entire context. In contrast, NLM averages pixels with similar values independently from the segmentation, preserving the missed text. As a result, this text is retained as if it had been accurately segmented by the algorithms, even if it smooths the results. Figure 15 shows how the methods that we tested perform on a good segmentation mask and on a bad segmentation mask.

**Figure 16:**
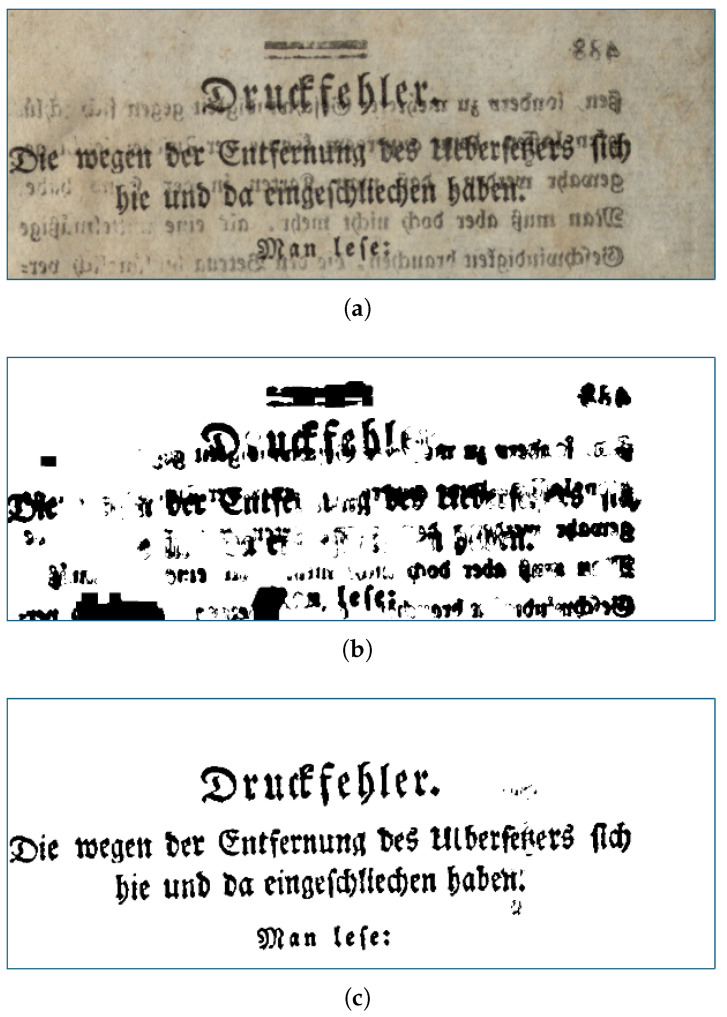
(**a**) The starting image. (**b**) The output mask obtained from the segmentation model without fine-tuning. (**c**) The output mask obtained from the segmentation model with fine-tuning.

Most of the bleed-through removal methods are applicable just to the documents they are trained on. The reason is that the documents are very different in style, in the texture of the paper, in the ornaments, and in other characteristics. Our method succeeds in smoothing out even strong bleed-through if it is not a preponderant feature of the page (i.e., it is not present in the page as much as the real text); moreover, for particularly different types of documents a little fine-tuning is needed. Figure 16 shows how the segmentation model performs before and after fine-tuning on a dataset different from the one the model was trained on. The dataset used to fine-tune is a combination of the DIBCO (i.e., Document Image Binarization Contest, which is an annual benchmarking competition focused on evaluating the performance of binarization methods applied to document images) images.

### 6.2. Model Selection Strategies

The pages of different manuscripts can vary significantly. For instance, one manuscript may suffer from severe ink fading without any bleed-through, while another may exhibit the opposite issue or both. Some manuscripts may contain large figures that bleed through to the reverse side, rendering the underlying text unreadable, whereas others may not contain any figures at all. Given these differences, it is clear that there is no universally optimal model suitable for all scenarios. The non-local means (NLM) method is a strong candidate in most cases, as it performs well in the presence of bleed-through, ink fading, when both issues occur simultaneously, and with lighting variations. However, selecting appropriate parameters is essential for optimal performance, as explained in Section 6.1. In general, NLM is a reliable and effective choice, but there are specific situations where alternative methods such as biweight or median inpainting can yield better results. Biweight estimation is particularly suitable when ink fading is severe and even a “weak” NLM configuration fails to preserve useful information. An illustrative example is shown in Figure 14. However, it does not perform well in cases with lightning variations and its results are artificial. On the other hand, the median inpainting method is recommended when ink fading and the lightning variations are not present and background texture is not a concern. This is because any pixel not classified as meaningful text or image is replaced with a uniform value, resulting in a clean, flat background.

### 6.3. Qualitative and Quantitative Results

We evaluate our results with two quantitative analyses on the segmentation masks generated from the DIBCO dataset. Specifically, we built a dataset with all the images of the DIBCO competitions from 2013 to 2018. With all this processing completed, we obtained a dataset of 826 patches. This is the dataset we used to fine-tune the text segmentation algorithm. In contrast, the ornament segmentation algorithm is fine-tuned on a simple dataset with all black masks because the DIBCO images have no ornaments. The page segmentation algorithm is not used because in the DIBCO images there is just the page and so there is no need to filter them.

The first quantitative analysis we make is about the foreground and the background error in the segmentation mask. The foreground error, in an image, is the number of foreground pixels that are wrongly classified as background over the number of foreground pixels (first equation of (13)). The background error, in an image, is the number of background pixels that are wrongly classified as foreground over the number of background pixels (second equation of (13)). Finally, the weighted total error is calculated as the weighted sum of the foreground and background errors (third equation of (13)), where the weights correspond to the ratio of background pixels in the entire image (for the background error) and the ratio of foreground pixels in the entire image (for the foreground error).

(13) $$\begin{array}{c} \mathrm{FgError}=\frac{1}{N_{Fg}}\sum_{t\in GT(Fg)}\left|GT\left(t\right)-B\left(t\right)\right|\\ \mathrm{BgError}=\frac{1}{N_{Bg}}\sum_{t\in GT(Bg)}\left|GT\left(t\right)-B\left(t\right)\right|\\ \mathrm{WTotError}=\frac{N_{Fg}\mathrm{FgError}+N_{Bg}\mathrm{BgError}}{N}. \end{array}$$

where *GT*(*t*) and *B*(*t*) are the ground truth and the predicted binarized mask for the specific pixel *t*, $N_{Fg}$ is the number of foreground pixels, $N_{Bg}$ is the number of background pixels, *GT*(*Fg*) represents the foreground area of the ground truth made of $N_{Fg}$ pixels, *GT*(*Bg*) is the background area of the ground truth constituted of $N_{Bg}$ pixels, and *N* is the total number of pixels.

**Figure 17:**
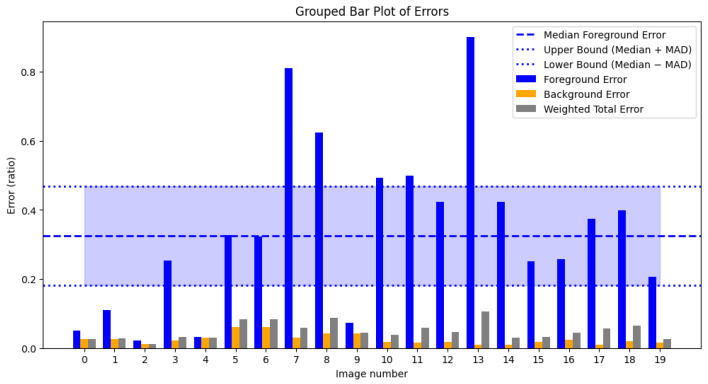
Foreground, background and weighted total errors for the 20 images of the DIBCO 2019 catalog.

**Figure 18:**
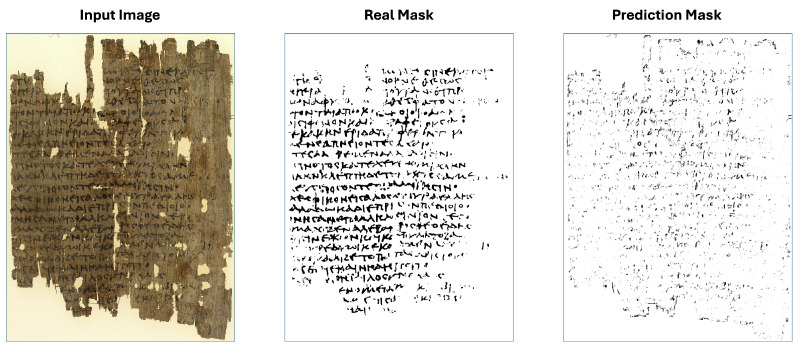
Input image index 13 with its real mask and its prediction mask.

**Figure 19:**
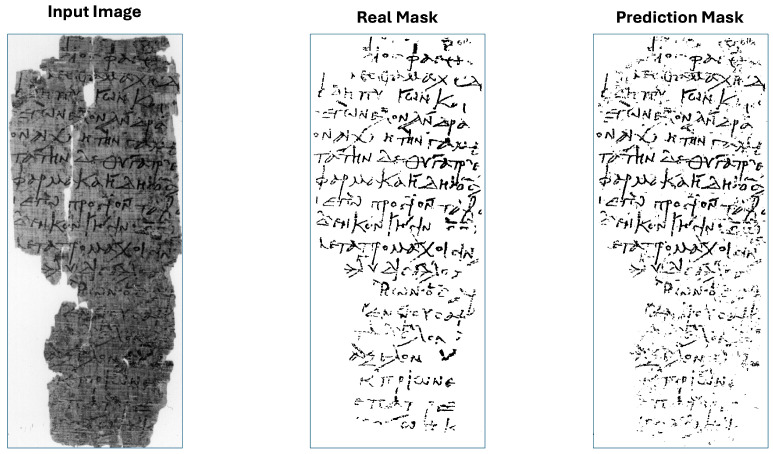
Input image index 16 with its real mask and its prediction mask.

The plot in Figure 17 indicates that the primary source of error is in the foreground, meaning that the model occasionally misclassifies foreground pixels as background in certain images. Moreover, even if the foreground error for some image is high, the Median Absolute Deviation (MAD) of the considered images is just 0.14, meaning that the mean distance of the foreground errors to their median (0.32) is small. So, our segmentation algorithm is consistent, with most foreground error around 0.32±0.14. While our model fails on some of the most degraded images, such as in Figure 18, it successfully handles others, even those on which it has not been explicitly trained, as in Figure 19.

Figure 18 shows how our segmentation models perform on the image with index 13 of DIBCO 2019 catalog.

Figure 19 shows how our segmentation models perform on the image with index 19 of DIBCO 2019 catalog.

The second quantitative analysis consists of calculating the F1-score, the Pseudo F1-score introduced by [37], the Peak Signal-to-Noise Ratio (PSNR), and the Distance-Reciprocal Distortion (DRD) presented by [38] on the images and then compare the results with the ones in [33].

The F1-score in Formula (14) is the harmonic average between recall and precision. Recall measures the proportion of actual foreground pixels that have been correctly identified, while precision quantifies the proportion of predicted foreground pixels that are truly part of the foreground. We want the precision and the recall to be as high as possible, even if usually there is a trade-off between these two.

(14) $$\begin{array}{c} F1\text{-}\mathrm{score}=2\times \frac{\mathrm{Precision}\times \mathrm{Recall}}{\mathrm{Precision}+\mathrm{Recall}}\\ \mathrm{Precision}=\frac{\mathrm{True}\;\mathrm{Positive}}{\mathrm{True}\;\mathrm{Positive}+\mathrm{False}\;\mathrm{Positive}}\\ \mathrm{Recall}=\frac{\mathrm{True}\;\mathrm{Positive}}{\mathrm{True}\;\mathrm{Positive}+\mathrm{False}\;\mathrm{Negative}} \end{array}$$

**Figure 20:**
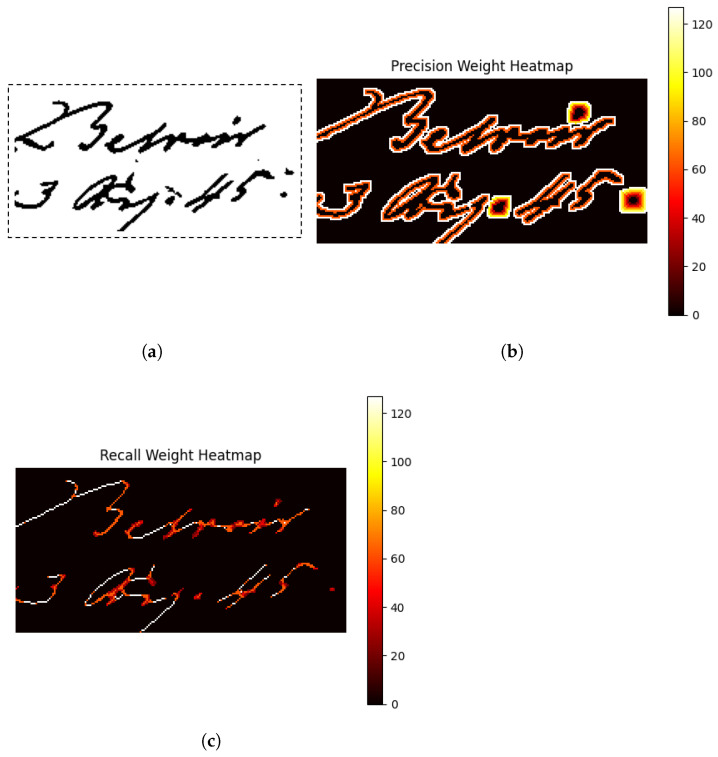
(**a**) The binary mask. (**b**) The weight mask of the precision. (**c**) The weight mask of the recall.

The Pseudo F1-score has the same formulation of the simple F1-score, but uses pseudo-precision and pseudo-recall. These two estimators are obtained by weighting the simple precision and recall metrics with respect to the weight masks. Figure 20 shows the weight masks. Pseudo-precision measures the amount of introduced noise and is different from the simple precision because the errors close to the borders of the text are more important than the others. Pseudo-recall measures the loss of information and is different from simple recall because within a letter, the error is more important if it is far from the contours and so the errors close to the borders are less important. The reason for using these masks is to assign different levels of importance to different pixels. The idea is that some pixels contribute more to the final evaluation of the binarization quality due to their relevance to the document’s content. More details about pseudo-precision and pseudo-recall can be found in [37].

PSNR measures the similarity between the binarized image and the ground truth mask based on pixel intensity. A higher PSNR indicates better similarity (a PSNR of around 20 suggests good results).

(15) $$\begin{array}{c} \mathrm{PSNR}=10\times \log_{10}\left(\frac{MAX^{2}}{MSE}\right) \end{array}$$

where *MAX* is the maximum pixel value (usually 255) and *MSE* is the Mean Square Error of the binarized and the ground truth mask pixels.

DRD measures the difference between two images trying to mimic the human visual system. Equations (16) show how to obtain the m×m normalized weight matrix, $W_{N}\left(i,j\right)$, centered in $i_{C}=j_{C}=\frac{\left(m+1\right)}{2}$, where *m* refers to the size of the window used to define the weight matrix $W_{N}\left(i,j\right)$. This normalized weight matrix penalizes distortions close to the edges and down-weights the distortions in smooth regions. Once this weight matrix is calculated, we must calculate $D_{k}\left(i,j\right)$ (first equation of (17)), where *k* is one of the *S* pixels wrongly classified in the predicted mask, $I{\left(i,j\right)}_{k}$ is the intensity of the pixel *k* at position (i,j) in the predicted mask, and $I_{GT}{\left(i,j\right)}_{k}$ is the intensity of the pixel *k* at position (i,j) in the ground truth mask. If $D_{k}\left(i,j\right)>0$, we weight $D_{k}\left(i,j\right)$ according to $W_{N}\left(i,j\right)$ and we obtain $DRD_{k}$ (second equation in (17)). To get the final *DRD* value we sum all the $DRD_{k}$ and divide the result for *NUBN*, which is the number of the non-uniform (not all black or white pixels) 8×8 blocks in the ground truth image.

(16) $$\begin{array}{c} W(i,j)=\left\{\begin{array}{cc} 0, & \mathrm{if}\;i=i_{C}\;\mathrm{and}\;j=j_{C},\\ \frac{1}{\sqrt{{\left(i-i_{c}\right)}^{2}+{\left(j-j_{C}\right)}^{2}}}, & \mathrm{otherwise}. \end{array}\right.\\ W_{N}\left(i,j\right)=\frac{W(i,j)}{\sum_{i=1}^{m}\sum_{j=1}^{m}W\left(i,j\right)}. \end{array}$$

(17) $$\begin{array}{c} D_{k}\left(i,j\right)=\left|I{\left(i,j\right)}_{k}-I_{GT}{\left(i,j\right)}_{k}\right|\\ DRD_{k}=\sum_{i,j}\left[D_{k}\left(i,j\right)*W_{N}\left(i,j\right)\right]\\ DRD=\frac{\sum_{k=1}^{S}DRD_{k}}{NUBN} \end{array}$$

The fine-tuned text and ornament segmentation models are then evaluated on the DIBCO 2019 images.

In Table 1, we present the results of the winner method (rank 1) and three of the other submitted methods to the DIBCO challenge of 2019 (i.e., rank 5, rank 10 and rank 20). We perform slightly worse than the 5th, way better than the 10th and the 20th methods, and our DRD is better than the one obtained by the winner.

**Figure 21:**
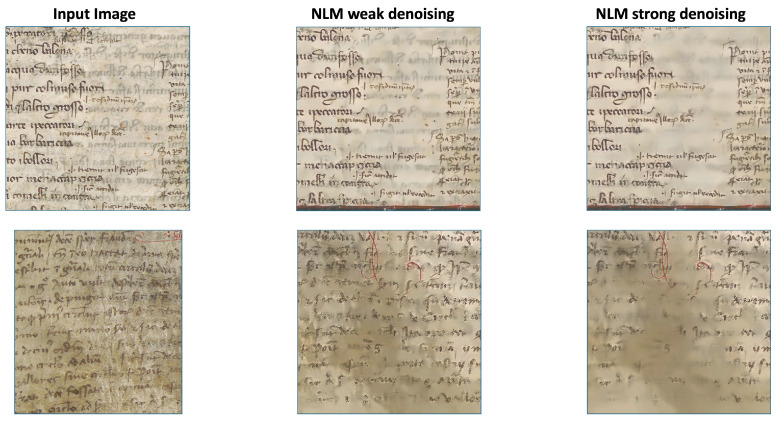
Two examples of application of our model. The first column shows the two input images, the second column is dedicated to the results of our model with the “weak” NLM (i.e., NLM with parameters h = 6, hColor = 20, templateWindowSize = 15, searchWindowSize = 35), and the third column shows the results of our model with the “strong” NLM (i.e., NLM with parameters h = 8, hColor = 20, templateWindowSize = 25, searchWindowSize = 55).

The first row of Figure 21 illustrates a case where bleed-through is the only degradation affecting the input image. In this case, the NLM “weak” filter enhances clarity by making the bleed-through faint and barely visible, while the NLM “strong” filter removes it entirely. In contrast, the second row presents a different degradation issue: ink fading. This occurs when the ink on the front page begins to vanish, making it resemble bleed-through. The NLM “strong” filter is ineffective in this scenario, as it removes the entire faded ink area. However, the NLM “weak” filter slightly smooths the image while preserving its most important features.

### 6.4. Inference Time, with GPU Comparison

One more aspect to consider is the speed of our model in minimizing the bleed-through effect. In Table 2, we show the average time required for segmentation, performed solely by the GPU (GPU time) and the total time needed to complete the entire bleed-through removal process (TOTAL elapsed time). The GPU time is included as part of the TOTAL elapsed time. Notice that the GPU acceleration is used just for segmentation, because the NLM inpainting is entirely performed by the CPU.

The tests were carried out on 111 JPEG images, each with an average size of 1348 pixels in width, 2073 pixels in height, and 3 channels. The average file size with JPEG compression is about 400 KB, whereas the average raw pixel data occupies 8 MB. Considering that each channel (red, green, blue) of these images uses 1 byte (8 bits) per pixel, the average raw pixel data can be calculated as follows: 1348×2073×3×1B=8,383,212B≈8MB. The devices used for these tests are heterogeneous, including workstation-class GPUs such as the NVIDIA RTX A4000 (with 16 GB of GDDR6 VRAM) and NVIDIA RTX 3090 (with 24 GB of GDDR6X VRAM), data center-class GPUs such as the L40S (with 48 GB of GDDR6 VRAM), the NVIDIA Tesla V100 (with 32 GB G HBM2 VRAM), and the NVIDIA H100 (with 80 GB HBM3 VRAM), and also a CPU Intel (R) Core(TM) i9-149000KF alone. The patches are processed in batches, with the batch size determined by the available VRAM capacity. To optimize computation, we maximized VRAM utilization.

It is important to note that the results can be further optimized, as the inference was performed on individual images. Specifically, the inference code used for testing takes advantage of GPU parallelization by dividing each image into square patches with a width of 400 pixels and a stride of 100 pixels, resulting in approximately 170 patches per image. These patches are processed in parallel. To further optimize performance, we can leverage this parallelization capability by feeding multiple images in bulk into the inference code.

Considering that a medieval manuscript can have 1000 pages, the time required to minimize the bleed-through effect for the whole book with a workstation equipped with an L40S GPU and an Intel i9-14900KF CPU is 1 h.

## 7. Conclusions

We propose an automatic blind method for restoring ancient manuscripts affected by bleed-through degradation. The main novelty of this research lies in the application of machine learning models to minimize the bleed-through effect in ancient manuscripts, making them readable even for non-experts. Our goal was to identify the most versatile and generalizable methods for this task, rather than focusing solely on those that achieve the best results on the specific manuscripts we analyzed. The main limitation of our model is that it minimizes the bleed-through effect without reconstructing the background. However, this drawback is actually advantageous, as it prevents the complete removal of valid text that might otherwise be mistakenly classified as bleed-through and so deleted during the background reconstruction phase.

Each image is segmented into foreground and background pixels, with bleed-through artifacts classified as part of the background. After binarization and cleaning using thresholding techniques and morphological operations, we obtain the final mask. We evaluate this mask by comparing our results with those achieved by competitors in the DIBCO competitions, demonstrating that our approach competes with top-performing models.

Finally, we apply inpainting to the background areas using non-local means (NLM), which reduces the bleed-through effect. The results show a significant improvement in degraded regions. However, with the “weak” NLM settings, while readability is enhanced, some bleed-through stains remain visible.

Future improvements could focus on enhancing the segmentation process, as bleed-through is sometimes misclassified as foreground text, causing it to remain visible in the cleaned image.

## Figures and Tables

**Table 1:** Evaluation results for some of the methods submitted to DIBCO 2019 and our method.

Method	F1	Pseudo F1	PSNR	DRD
rank 1	72.87	72.15	14.475	16.71
rank 5	62.985	61.01	14.32	10.84
Ours	61.83	60.44	13.58	12.19
rank 10	60.145	56.7	11.745	36.52
rank 20	46.63	44.06	13.09	15.57

**Table 2:** This table compares the performance of different GPUs and one CPU in terms of time for segmentation and total processing time. The shown results represent the average time of five runs. Notice that the CPU used in the workstation of the NVIDIA L40S is the Intel i9-14900KF, so with using parallelization capabilities of the GPU we perform the same computation in 5% of the time needed by the CPU alone. The workstations of the GPUs are equipped with different CPUs and storages, that is why the H100 has an higher TOTAL time than the L40S despite having a smaller GPU time.

Name	GPU Time (Segmentation) (s)	TOTAL Elapsed Time (s)
NVIDIA H100 (GPU)	1.00	4.76
NVIDIA L40S (GPU)	1.93	3.60
NVIDIA 3090 (GPU)	2.06	3.65
NVIDIA V100 (GPU)	3.19	7.91
NVIDIA A4000 (GPU)	4.10	8.73
Intel i9-149000KF (CPU)	NaN	62.70

## Data Availability

All data used and referenced in this work are publicly available on the official website of the MAGIC project: https://www.magic.unina.it/.
